# Does travel distance influence short‐term outcome after periacetabular osteotomy?—A cohort study

**DOI:** 10.1002/jeo2.70912

**Published:** 2026-09-24

**Authors:** Quentin Karisch, Chiara Heller, Justus Stamp, Marco Haertlé, Henning Windhagen, Sufian S. Ahmad

**Affiliations:** ^1^ Department of Orthopedics Hannover Medical School Hannover Germany

**Keywords:** distance, outcome, patient‐reported outcome measures (PROM), periacetabular osteotomy (PAO), travel

## Abstract

**Purpose:**

Periacetabular osteotomy (PAO) is an established joint‐preserving procedure for the treatment of developmental dysplasia of the hip (DDH). As PAO is typically concentrated in specialized centres, many patients travel considerable distances for treatment. The purpose of this study was to evaluate the influence of travel distance on short‐term outcomes following PAO.

**Methods:**

This prospective registry study included 296 hips undergoing PAO for DDH between January 2022 and May 2025 by a single experienced surgeon. Travel distance was determined using driving distance, and hips were stratified into two groups: ≤80.5 and >80.5 km from the treating centre. Radiographic parameters, patient‐reported outcome measures (PROMs) and rates of achieving the Patient Acceptable Symptom State (PASS) and Minimal Clinically Important Difference (MCID) for the modified Harris Hip Score (mHHS) and international Hip Outcome Tool‐12 (iHOT‐12) were analysed.

**Results:**

A total of 126 hips (43%) resided ≤80.5 km and 170 hips (57%) >80.5 km from the treating centre. Hips of patients travelling longer distances demonstrated a lower preoperative lateral centre‐edge angle (*p* = 0.005) and a higher extrusion index (*p* = 0.034). No significant differences were observed in demographics, comorbidities, concomitant procedures or preoperative PROMs. All PROMs improved significantly in both groups (all *p* < 0.05). Postoperative PROMs, magnitude of improvement, PASS and MCID achievement rates and complication rates did not differ significantly between groups. Travel distance was not an independent predictor of postoperative outcomes in multivariable regression analyses.

**Conclusion:**

Travel distance to a specialized hip preservation centre does not influence short‐term outcomes following PAO. Comparable functional outcomes and complication rates can be achieved irrespective of travel distance, supporting the feasibility of centralized PAO care in specialized high‐volume centres.

**Level of Evidence:**

Level III.

## Graphical abstract

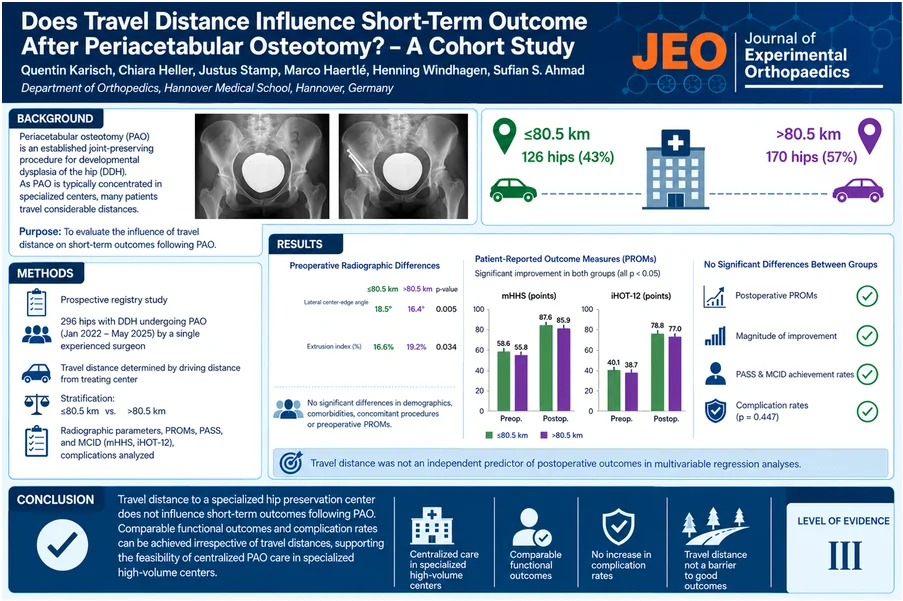

**Abbreviations**
AC: anterior wall coverage
AI: acetabular index
BMI: body mass index
DDH: developmental dysplasia of the hip
EI: extrusion index
G‐FJS: German Forgotten Joint Score
HHS: Harris Hip Score
HOOS‐PS: Hip Disability and Osteoarthritis Outcome Score—Physical Function Shortform
iHOT‐12: international Hip Outcome Tool‐12
km: kilometre
LCEA: lateral centre‐edge angle
MCID: Minimal Clinically Important Difference
mHHS: modified Harris Hip Score
mi: miles
PAO: periacetabular osteotomy
PASS: Patient Acceptable Symptome State
PC: posterior wall coverage
PMA: Postel Merle d'Aubigné Score
PROM: patient‐reported outcome measure
SD: standard deviation
UCLA: University of California and Los Angeles Activity Scale
WOMAC: Western Ontario and McMaster Universities Osteoarthritis Index

## INTRODUCTION

Developmental dysplasia of the hip (DDH) is characterized by insufficient acetabular coverage of the femoral head. Periacetabular osteotomy (PAO) is an established and effective joint‐preserving treatment for symptomatic DDH in adults [1, 15, 31, 33]. Owing in part to the widespread implementation of neonatal screening programs, the prevalence of DDH has declined to approximately 2.3% [16, 29]. Consequently, patients often need to travel considerable distances to access specialized centres offering PAO.

For technically demanding procedures such as PAO, surgeon experience and procedural volume are critical determinants of outcome. Previous studies have demonstrated a learning curve ranging from 26 to 50 PAOs before improvements in operative time, blood loss, accuracy of correction and complication rates are achieved [17, 24, 28]. Furthermore, maintaining proficiency has been associated with an annual case volume of approximately 50 PAOs [17].

More broadly, treatment in high‐volume specialized centres has been linked to superior outcomes across various surgical disciplines. Higher procedural volumes have been associated with lower mortality rates, improved postoperative outcomes, reduced revision rates, fewer complications such as infections, shorter hospital stays and lower healthcare costs [9, 26, 35]. In hip preservation surgery, PAO is increasingly concentrated in specialized referral centres because of its technical complexity and the importance of surgical expertise [31].

Patients with more complex conditions are generally more willing to travel longer distances to receive specialized care [11, 14, 30]. However, the impact of travel distance on surgical outcomes remains controversial. While several studies have reported higher complication rates among patients travelling greater distances [10, 11, 30], others have found no association between travel distance and postoperative complications [14, 21, 22].

Evidence regarding the association between travel distance and patient‐reported outcomes remains limited. Existing studies have primarily focused on patients undergoing total joint arthroplasty [21, 22] or treatment for femoroacetabular impingement syndrome [6].

Continuity of care may represent an additional factor influencing postoperative outcomes. Transfer to or readmission at a different hospital has been associated with increased postoperative mortality rates [38].

Although substantial improvements in patient‐reported outcome measures (PROMs) following PAO have been consistently reported [23, 24], recent studies have further highlighted the relevance of patient characteristics and acetabular morphology for patient‐reported outcomes following PAO [18, 24]. However, no study has investigated the relationship between travel distance to a specialized hip preservation centre and clinical outcomes, including PROMs, following PAO for DDH.

Given these potentially opposing effects of travel distance on surgical outcomes, the aim of the present study was to evaluate the association between travel distance and short‐term outcomes following PAO [3]. This question is of particular interest because PAO is predominantly performed in a young patient population with generally low rates of complications, although such adverse events may have long‐term consequences over a substantial remaining lifespan [8, 20, 34]. Furthermore, patients willing to travel longer distances may differ systematically in their treatment expectations, motivation and healthcare‐seeking behaviour.

It was hypothesized that patients travelling >50 miles (mi)/80.5 kilometres (km) to undergo PAO would demonstrate superior short‐term outcomes compared with patients residing closer to the treating centre.

## METHODS

**Figure 1:**
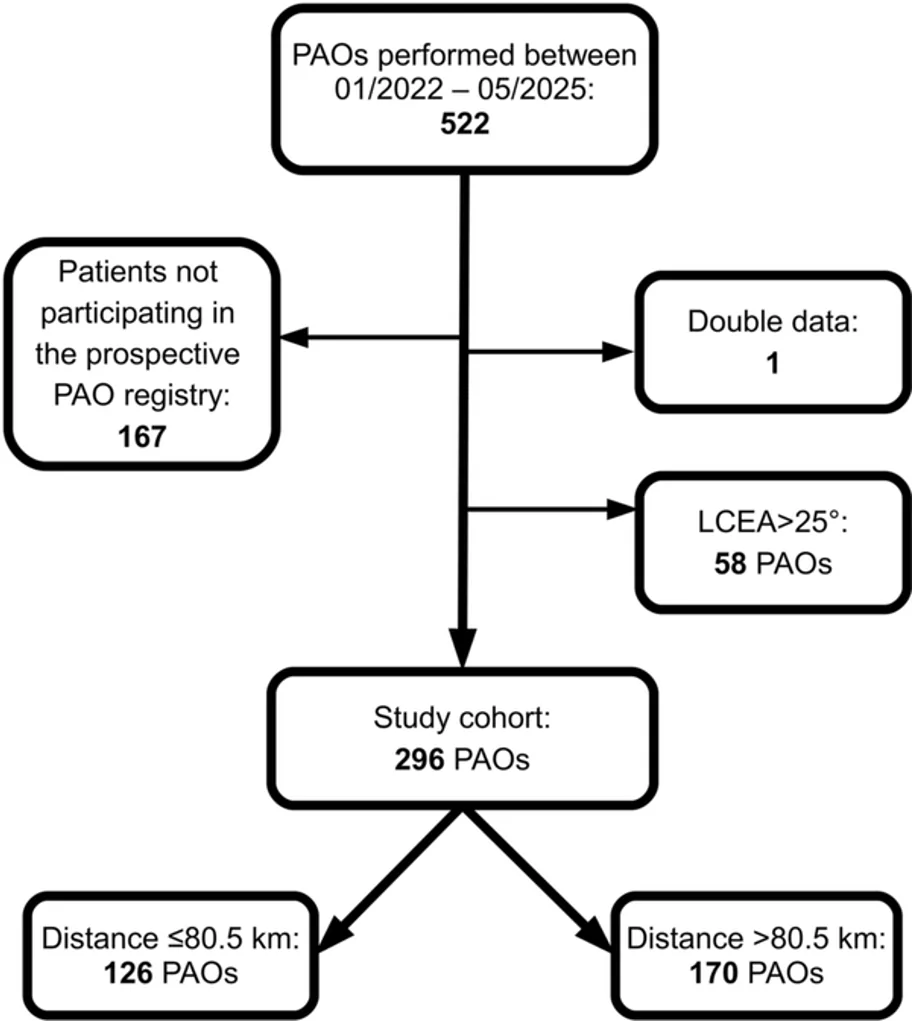
Flowchart demonstrating the selection process of hips included in the final analysis. km, kilometre; LCEA, lateral centre‐edge angle; PAOs, periacetabular osteotomies.

A prospective PAO registry was used for this study. Between January 2022 and May 2025, a total of 522 PAOs were performed by a single high‐volume hip preservation surgeon. Of these, 296 hips of patients who consented to participation in the registry, including a planned 10‐year follow‐up and met the study eligibility criteria (Figure 1).

Hips were eligible if they underwent PAO for symptomatic DDH, defined by a lateral centre‐edge angle (LCEA) < 25°. Patients were excluded if language barriers prevented reliable completion of the PROM questionnaires.

Hips were stratified according to the travel distance between their place of residence and the treating centre. Travel distance was determined using the driving route calculated by OpenStreetMap (OpenStreetMap Foundation) and recorded in kilometres. Accordingly, two groups were defined: ≤80.5 and >80.5 km. A distance of 80.5 km was considered equivalent to 50 miles. The 80.5 km cutoff was selected a priori based on its use in previous studies investigating the association between travel distance and outcomes after orthopaedic surgery, including hip preservation surgery [6, 22]. This threshold was chosen to facilitate comparability with the existing literature and should be considered a pragmatic, literature‐based threshold rather than a clinically validated cutoff for patients undergoing PAO.

### Radiographic assessment

All hips underwent standardized radiographic evaluation according to the institutional PAO protocol, including a preoperative anteroposterior pelvic radiograph. Hip morphology was assessed using the following radiographic parameters: LCEA, acetabular index (AI), extrusion index (EI), anterior wall coverage (AC) and posterior wall coverage (PC). All radiographic measurements were performed by experienced orthopaedic surgeons according to the methodology described by Tannast et al. [36].

### PROMs and clinical assessment

The following validated PROMs were collected: University of California and Los Angeles activity scale (UCLA), Hip Disability and Osteoarthritis Outcome Score—Physical Function Shortform (HOOS‐PS), Western Ontario and McMaster Universities Osteoarthritis Index (WOMAC), German Forgotten Joint Score (G‐FJS), international Hip Outcome Tool‐12 (iHOT‐12), Harris Hip Score (HHS), modified Harris Hip Score (mHHS) and Postel Merle d'Aubigné (PMA) score. The iHOT‐12 and mHHS were considered the key PROMs for the assessment of clinically meaningful outcomes, as validated Patient Acceptable Symptom State (PASS) and Minimal Clinically Important Difference (MCID) thresholds were available for these instruments. The following thresholds were applied: PASS ≥ 71 [37] and MCID ≥ 8 [25] for the mHHS and PASS ≥ 44 [4] and MCID ≥ 13 [27] for the iHOT‐12. PROM questionnaires were completed independently by the patients. Clinical components of the HHS were assessed and documented by trained medical personnel.

PROMs were collected preoperatively and at 12, 24 and 36 months postoperatively. The HHS was recorded at 12 and 24 months postoperatively. For the present analysis, the longest available follow‐up for each hip was used.

Postoperative complications were recorded from institutional medical records and patient‐reported questionnaires sent during follow‐up. Complications treated at external institutions were captured when reported by the patients.

In addition, concomitant procedures performed during PAO and pre‐existing comorbidities were recorded.

### Variables and endpoints

The primary endpoint was the comparison of postoperative PROMs between hips of patients travelling ≤80.5 km and those travelling >80.5 km to undergo PAO at short‐term follow‐up.

Secondary endpoints included rates of achieving PASS and MCID, perioperative characteristics and intraoperative and postoperative complications.

### Statistical analysis

Data collection was performed using Microsoft Excel (Microsoft Corp.). Statistical analyses were conducted using IBM SPSS Statistics Version 30.0.0.0 (IBM Corp.). Figures were generated using GraphPad Prism version 11 (GraphPad Software). An a priori power analysis was performed using G*Power (Version 3.1.9.7). Assuming a moderate effect size and a statistical power of 80%, a minimum of 64 hips per group was required to detect a statistically significant between‐group difference. ChatGPT (OpenAI) was used for language editing and optimization of grammar, syntax and linguistic clarity and for the creation of the visual abstract. The authors reviewed and approved all changes and take full responsibility for the content accuracy and integrity of the manuscript and visual abstract.

Continuous variables were assessed for normality using the Shapiro‐Wilk test and are presented as mean with standard deviation (SD). Homogeneity of variances was evaluated using Levene's test. Categorical variables are presented as frequencies and percentages.

For between‐group comparisons, an independent‐samples *t* test was used for normally distributed variables with homogeneous variances, whereas Welch's *t* test was applied when variances were unequal. Non‐normally distributed continuous variables were analysed using the Mann–Whitney *U* test. Categorical variables were compared using Fisher's exact test.

For within‐group comparisons, paired *t* tests were used for normally distributed variables and Wilcoxon signed‐rank tests for non‐normally distributed variables. Effect sizes were calculated for all primary comparisons.

To account for potential baseline heterogeneity between groups, multivariable linear regression analyses were performed for each PROM at final follow‐up. The corresponding preoperative PROM was included as an independent covariate in each model to adjust for baseline differences. Furthermore, all models were adjusted for travel‐distance group and demographic variables that differed significantly between groups preoperatively. As a sensitivity analysis, multivariable regression analyses were repeated with travel distance modelled as a continuous variable rather than as a dichotomous variable. A two‐sided *p* value < 0.05 was considered statistically significant.

## RESULTS

### Baseline characteristics

Preoperatively, the groups differed significantly only with respect to radiographic measures of dysplasia severity. Hips of patients travelling >80.5 km demonstrated a lower mean LCEA (15.74° vs. 17.53°, *p* = 0.005) and a higher mean EI (30.06% vs. 28.59%, *p* = 0.034) compared with hips of patients travelling ≤80.5 km.

No significant differences were observed between groups regarding age, body mass index (BMI), sex, operative side, number of concomitant procedures performed during PAO, previous surgical procedures, comorbidities, AI, AC or PC (all *p* > 0.05) (Table 1).

**Table 1:** Descriptive data: Dysplasia in hips of patients with distance ≤/>80.5 km.

	≤80.5 km		>80.5 km		*p* value	Effect size
	*N*	Mean ± SD	*N*	Mean ± SD		
Number of hips						
		126/296 (43%)		170/296 (57%)		
Average age (years)						
	126	30.82 ± 8.65	170	28.88 ± 7.88	0.056^b^	−0.111
Body mass index (kg/m^2^)						
	126	25.71 ± 5.19	166	24.80 ± 4.66	0.213^b^	−0.073
Sex						
Male		25/126 (20%)		24/170 (14%)	0.208^a^	0.076
Female		101/126 (80%)		146/170 (86%)		
Side						
Right		63/126 (50%)		93/170 (55%)	0.480^a^	0.047
Left		63/126 (50%)		77/170 (45%)		
Additional intervention during PAO						
Yes		28/126 (22%)		33/170 (19%)	0.564^a^	0.034
No		98/126 (78%)		137/170 (81%)		
Previous operation						
Yes		12/103 (12%)		11/130 (8.5%)	0.508^a^	0.053
No		91/103 (88%)		119/130 (91.5%)		
Comorbidity						
Yes		34/104 (33%)		41/130 (31.5%)	0.888^a^	0.012
No		70/104 (67%)		89/130 (68.5%)		
Preoperative radiographic measurements						
LCEA (°)	126	17.53 ± 5.44	170	15.74 ± 5.84	**0.005** b	−0.164
AI (°)	126	14.40 ± 5.84	170	15.67 ± 6.49	0.104^b^	−0.094
EI (%)	126	28.59 ± 6.26	170	30.06 ± 6.30	**0.034** b	−0.123
AC (%)	126	40.24 ± 14.82	170	38.29 ± 12.78	0.281^b^	−0.063
PC (%)	126	83.21 ± 17.52	170	83.38 ± 19.06	0.938^c^	−0.009

*Note*: Effect sizes: *φ* for Fisher's exact test; Cohen's *d* for unpaired *t* test; $r=Z/\sqrt{N_{\text{total}}}$ for Mann–Whitney *U*. CI (Cohen's *d*)_PC_: [−0.240; 0.221]; bold *p* values indicate statistical significance (*p* < 0.05).

Abbreviations: AC, anterior wall coverage; AI, acetabular index; CI, 95% confidence interval; EI, extrusion index; km, kilometre; LCEA, lateral centre‐edge angle; PC, posterior wall coverage; SD, standard deviation.

^a^ Fisher's exact test.

^b^ Mann–Whitney *U* test.

^c^ Unpaired *t* test.

**Figure 2:**
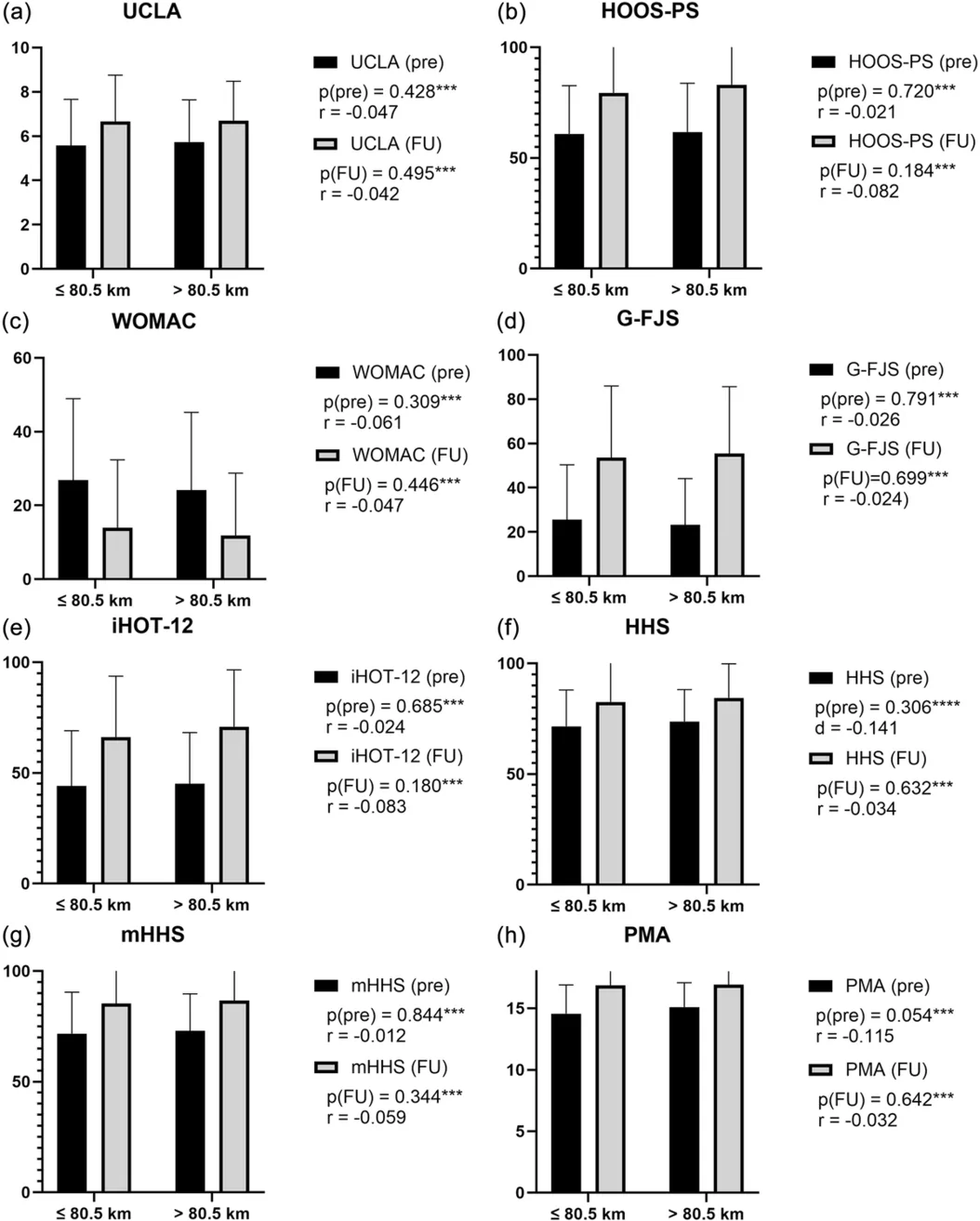
(a) Comparison of pre and FU on the UCLA between hips of patients with distance ≤80.5 km (meanpre = 5.58, SDpre = 2.08, meanFU = 6.67, SDFU = 2.08, **p < 0.001, *r* = −0.591) and hips of patients with distance >80.5 km (meanpre = 5.74, SDpre = 1.90, meanFU = 6.70, SDFU = 1.78, **p < 0.001, *r* = −0.478). (b) Comparison of pre and FU scores on the HOOS‐PS between hips of patients with distance ≤80.5 km (meanpre = 60.87, SDpre = 21.72, meanFU = 79.39, SDFU = 20.71, *p < 0.001, *d* = −0.784, CI (Cohen's *d*): [−1.001; −0.563], CI (MD): [−21.305; −12.867]) and hips of patients with distance >80.5 km (meanpre = 61.71, SDpre = 21.96, meanFU = 83.07, SDFU = 17.67, *p < 0.001, *d* = −0.974, CI (Cohen's *d*): [−1.169; −0.776], CI (MD): [−24.150; −17.224]). (c) Comparison of pre and FU scores on the WOMAC between hips of patients with distance ≤80.5 km (meanpre = 26.90, SDpre = 22.01, meanFU = 13.94, SDFU = 18.43, **p < 0.001, *r* = −0.548) and hips of patients with distance >80.5 km (meanpre = 24.14, SDpre = 21.09, meanFU = 11.80, SDFU = 16.99, **p < 0.001, *r* = −0.667). (d) Comparison of pre and FU scores on the G‐FJS between hips of patients with distance ≤80.5 km (meanpre = 25.65, SDpre = 24.73, meanFU = 53.63, SDFU = 32.31, *p = 0.042, *d* = −0.970, CI (Cohen's *d*): [−1.415; −0.512], CI (MD): [−44.165; −18.932]) and hips of patients with distance >80.5 km (meanpre = 23.23, SDpre = 20.88, meanFU = 55.42, SDFU = 30.19, *p = 0.002, *d* = −0.928, CI (Cohen's *d*): [−1.226; −0.624], CI (MD): [−35.127; −19.927]). (e) Comparison of pre and FU scores on the iHOT‐12 between hips of patients with distance ≤80.5 km (meanpre = 44.14, SDpre = 24.86, meanFU = 66.10, SDFU = 27.62, **p < 0.001, *r* = −0.603) and hips of patients with distance >80.5 km (meanpre = 45.04, SDpre = 23.16, meanFU = 70.91, SDFU = 25.66, **p < 0.001, *r* = −0.728). (f) Comparison of pre and FU scores on the HHS between hips of patients with distance ≤80.5 km (meanpre = 71.55, SDpre = 16.44, meanFU = 82.52, SDFU = 17.70, **p = 0.003, *r* = −0.603) and hips of patients with distance >80.5 km (meanpre = 73.72, SDpre = 14.42, meanFU = 84.36, SDFU = 15.45, **p < 0.001, *r* = −0.708) (CI (Cohen's *d*) HHSpre: [−0.411; 0.129]). (g) Comparison of pre and FU scores on the mHHS between hips of patients with distance ≤80.5 km (meanpre = 71.70, SDpre = 18.74, meanFU = 85.32, SDFU = 16.15, **p < 0.001, *r* = −0.626) and hips of patients with distance >80.5 km (meanpre = 72.93, SDpre = 16.77, meanFU = 86.60, SDFU = 15.11, **p < 0.001, *r* = −0.663). (h) Comparison of pre and FU scores on the PMA between hips of patients with distance ≤80.5 km (meanpre = 14.55, SDpre = 2.35, meanFU = 16.87, SDFU = 1.55, **p < 0.001, *r* = −0.774) and hips of patients with distance >80.5 km (meanpre = 15.10, SDpre = 1.97, meanFU = 16.91, SDFU = 1.62, **p < 0.001, *r* = −0.703). *Paired *t* test; **Wilcoxon test; *** Mann–Whitney *U* test; ****unpaired *t* test effect sizes (*r*): paired Cohen's *d* for paired *t* test; *r* $=Z/\sqrt{N_{\text{total}}}$ for Mann–Whitney *U*; $r=Z/\sqrt{N_{\text{pairs}}}$ for Wilcoxon signed‐rank test. CI, 95% confidence interval; FU, follow‐up; HHS, Harris Hip Score; HOOS‐PS, Hip Disability and Osteoarthritis Outcome Score—Physical Function Shortform; iHOT‐12, international Hip Outcome Tool—12; km, kilometre; MD, mean difference; mHHS, modified Harris Hip Score; Pre, preoperative; PMA, Postel Merle d'Aubigné score; *r*, effect size; SD, standard deviation; UCLA, University of California and Los Angeles activity scale; WOMAC, Western Ontario and McMaster Universities Osteoarthritis Index.

Similarly, no statistically significant differences in preoperative PROMs were identified between the two groups (all *p* > 0.05) (Figure 2).

### Within‐group and between‐group outcome comparison

Mean follow‐up duration ranged from 16.17 to 21.69 months (Table 2).

**Table 2:** Follow‐up duration and follow‐up rates in hips of patients with distance ≤/>80.5 km.

	Follow‐up duration (months)		Follow‐up rates (%)	
	≤80.5 km	>80.5 km	≤80.5 km	>80.5 km
UCLA				
	21.33	20.29	85.2	89.6
HOOS‐PS				
	21.33	20.24	85.4	89.6
WOMAC				
	21.42	20.34	84.0	88.3
G‐FJS				
	21.69	20.35	80.0	91.0
iHOT‐12				
	21.42	20.29	84.4	89.0
HHS				
	17.79	16.17	72.4	67.0
mHHS				
	21.33	20.32	85.6	87.9
PMA				
	17.87	17.09	73.6	72.3

Abbreviations: HHS, Harris hip score; HOOS‐PS, Hip Disability and Osteoarthritis Outcome Score—Physical Function Shortform; iHOT‐12, International hip outcome tool—12; km, kilometre; mHHS, Modified Harris hip score; PMA, Postel Merle d'Aubigné score; UCLA, University of California and Los Angeles activity scale; WOMAC, Western Ontario and McMaster Universities Osteoarthritis‐Index.

Follow‐up rates ranged from 67% to 91% (Table 2).

All PROMs demonstrated significant improvement from baseline to final follow‐up in both groups (all *p* < 0.05). However, no significant between‐group differences were observed regarding the magnitude of improvement for any PROM, including the key PROMs iHOT‐12 and mHHS (all *p* > 0.05) (Table 3).

**Table 3:** Differences between the PROMs preoperatively versus follow‐up at dysplasia in hips of patients with distance ≤/>80.5 km.

	≤80.5 km				>80.5 km				*p* value	Effect size
	*N*	*Δ* Preop versus FU (mean ± SD)			*N*	*Δ* Preop versus FU (mean ± SD)				
UCLA										
	104	0.96 ± 1.77			147	0.82 ± 1.92			0.791^a^	−0.017
HOOS‐PS										
	105	17.09 ± 21.80			147	20.69 ± 21.25			0.191^b^	−0.168
WOMAC										
	100	−11.82 ± 20.67			143	−11.43 ± 17.21			0.802^a^	−0.016
G‐FJS										
	28	31.55 ± 32.54			61	27.53 ± 29.67			0.566^b^	−0.131
iHOT‐12										
	103	21.17 ± 29.13			146	24.08 ± 24.67			0.412^a^	−0.052
HHS										
	71	12.90 ± 17.94			77	13.74 ± 14.85			0.758^c^	−0.051
mHHS										
	107	12.93 ± 18.82			145	12.40 ± 17.33			0.662^a^	−0.027
PMA										
	89	2.27 ± 2.43			115	1.79 ± 2.26			0.216^a^	−0.087

*Note*: $r=Z/\sqrt{N_{\text{total}}}$ for Mann–Whitney *U* CI (Cohen's *d*)_HOOs‐PS_: [−0.418; 0.083]; CI (Cohen's *d*)_G‐FJS_: [−0.317; 0.579]; CI (Cohen's *d*)_HHS_: [−0.374; 0.271].

Abbreviations: *Δ*, difference; CI, 95% confidence interval; FU, follow‐up; HHS, Harris hip score; HOOS‐PS, Hip Disability and Osteoarthritis Outcome Score—Physical Function Shortform; iHOT‐12, International hip outcome tool—12; km, kilometre; mHHS, Modified Harris hip score; PMA, Postel Merle d'Aubigné score; PROMs, patient‐reported outcome measures; SD, standard deviation; UCLA, University of California and Los Angeles activity scale; WOMAC, Western Ontario and McMaster Universities Osteoarthritis‐Index.

^a^ Mann–Whitney *U* test.

^b^ Unpaired *t* test.

^c^ Welch *t* test effect sizes: Cohen's *d* for unpaired and Welch *t* test.

Likewise, no significant differences were found between groups in the rates of achieving the PASS or the MCID for either the mHHS or iHOT‐12 (all *p* > 0.05) (Table 4).

**Table 4:** Rate of PASS and MCID for mHHS and iHOT‐12: Dysplasia in hips of patients with distance ≤/> 80.5 km.

	mHHS			iHOT‐12		
	≤80.5 km	>80.5 km	*p* value (effect size)	≤80.5 km	>80.5 km	*p* value (effect size)
PASS rate	*PASS mHHS* = *71*			*PASS iHOT‐12* = *44*		
Pre	74/125 (59%)	99/165 (60%)	0.904^a^ (0.008)	60/122 (49%)	77/164 (47%)	0.721^a^ (0.022)
Follow‐up	95/108 (88%)	127/150 (85%)	0.473^a^ (−0.047)	83/107 (78%)	126/152 (83%)	0.338^a^ (0.066)
MCID rate	*MCID mHHS* = *8*			*MCID iHOT‐12* = *13*		
	64/107 (60%)	88/145 (61%)	0.897^a^ (0.009)	66/103 (64%)	99/146 (68%)	0.587^a^ (0.039)

*Note*: Effect sizes: *φ* for Fisher's exact test.

Abbreviations: iHOT‐12, international hip outcome tool—12; km, kilometre; MCID, Minimal Clinically Important Difference; mHHS, modified Harris hip score; PASS, Patient Acceptable Symptom State; pre, preoperative.

a Fisher's exact test.

After adjustment for the corresponding preoperative PROM score and the preoperative radiographic parameters LCEA and EI, multivariable linear regression analyses showed no statistically significant association between travel‐distance group assignment and any postoperative PROM at final follow‐up. The estimated effects of travel‐distance group assignment were small for UCLA (*B* = −0.040, 95% CI −0.451 to 0.370; *p* = 0.847), HOOS‐PS (*B* = 3.095, 95% CI −1.250 to 7.441; *p* = 0.162), WOMAC (*B* = −0.141, 95% CI −3.975 to 3.693; *p* = 0.942), iHOT‐12 (*B* = 2.417, 95% CI −3.621 to 8.455; *p* = 0.431), HHS (*B* = −0.002, 95% CI −3.677 to 3.673; *p* = 0.999), mHHS (*B* = 0.068, 95% CI −3.446 to 3.582; *p* = 0.970), G‐FJS (*B* = −6.523, 95% CI −19.523 to 6.476; *p* = 0.321) and PMA (*B* = −0.105, 95% CI −0.550 to 0.340; *p* = 0.643). For the two key PROMs, iHOT‐12 and mHHS, the 95% confidence intervals for the adjusted travel distance effect remained entirely within the respective MCID thresholds, suggesting that any potential between‐group effect was unlikely to reach the predefined threshold for a clinically meaningful difference. The corresponding preoperative PROM remained a statistically significant predictor of postoperative outcome in all models (all *p* < 0.05) (Table S1).

Sensitivity analysis with travel distance modelled as a continuous variable yielded consistent results, with no significant association between travel distance and postoperative PROMs (Table S2).

### Complications

The overall complication rate was 8.0% (9/113) in the ≤80.5 km group and 5.1% (8/158) in the >80.5 km group. Complications in both groups (≤80.5/>80.5 km) included: implant‐related failure (2.7%/1.3%), delayed union (1.8%/0.6%), persistent symptoms (0.9%/0.0%), nerve‐related pathology (0.0%/0.6%), inflammation or infection (0.9%/1.3%), undercorrection (0.9%/0.0%), impingement (0.9%/0.6%) and other complications (0.0%/1.3%). No thromboembolic events were observed in either group. No statistically significant difference in overall complication rates was observed between the two groups (*p* = 0.447).

## DISCUSSION

The principal finding of the present study is that travel distance to a specialized hip preservation centre was not associated with poorer short‐term outcomes following PAO for DDH. Patients travelling more than 80.5 km (50 mi) demonstrated comparable preoperative PROMs, postoperative PROMs, rates of achieving the MCID and PASS and complication rates compared with patients residing closer to the treating centre. The consistency of these findings across multiple PROMs, including the key PROMs iHOT‐12 and mHHS, further supports the robustness of the results. Overall, both groups experienced substantial and clinically meaningful improvements following PAO.

The present study extends the existing literature on the relationship between travel distance and outcomes after orthopaedic procedures to patients undergoing PAO for DDH. Despite the specialized nature of PAO and the potential need for patients to travel considerable distances to access high‐volume hip preservation centres, travel distance was not associated with patient‐reported outcomes in the present cohort. Specifically, no significant differences were observed in preoperative or postoperative PROMs or in the achievement of PASS or MCID for the mHHS and iHOT‐12 and multivariable analyses adjusting for baseline differences did not identify an association between travel distance and postoperative PROMs. These findings suggest that patients travelling longer distances for PAO can achieve comparable patient‐reported outcomes to those living closer to the treating institution.

These findings are consistent with previous reports from other orthopaedic populations. In patients undergoing hip arthroscopy for femoroacetabular impingement syndrome, Beck et al. found lower preoperative HOS‐ADL, mHHS and iHOT‐12 scores among patients travelling more than 50 miles for surgery. However, these differences were no longer evident at 2 years postoperatively and neither MCID achievement nor multivariable analyses differed according to travel distance [6]. Similarly, Jones et al. reported no differences in preoperative or postoperative PROMs, MCID achievement or complication rates according to travel distance among patients undergoing total hip or knee arthroplasty [21, 22]. Consistent with these findings, complication rates following PAO did not differ significantly according to travel distance in the present study. The higher rate of 90‐day return visits to the treating institution among patients living closer to the hospital after total hip arthroplasty reported by Jones et al. could not be meaningfully assessed in the present cohort because routine postoperative follow‐up, including radiographic evaluation approximately 6 weeks after PAO, is an integral component of the institutional treatment protocol.

Importantly, this study specifically evaluates the role of travel distance in patients undergoing PAO for DDH, a highly specialized hip preservation procedure that may require referral to high‐volume centres. The absence of differences across patient‐reported outcomes, clinically meaningful improvement and complication rates suggests that geographic distance from a specialized centre should not be considered a determinant of expected outcome following PAO.

Previous literature has suggested that patients travelling greater distance for healthcare often present with more complex pathology [11, 14, 30]. In the setting of PAO, disease complexity may be reflected by factors such as the need for concomitant procedures, previous hip surgery or the presence of pre‐existing comorbidities. In the present cohort, however, no significant differences were observed in comorbidity burden, rates of previous surgery or the number of concomitant procedures performed at the time of PAO. The only baseline differences identified were a lower LCEA and a higher EI among patients travelling more than 80.5 km, indicating slightly more severe radiographic dysplasia. Such differences may reflect variation in the underlying morphological phenotype of symptomatic hip dysplasia, which has recently been characterized in greater detail [18]. One possible explanation is that patients residing farther from specialized hip preservation centres may experience delayed referral and therefore present with more advanced structural deformities. However, the clinical relevance of these relatively small radiographic differences appears limited, as they did not translate into differences in patient‐reported outcomes or complication rates.

The findings of the present study have important implications for the organization of hip preservation care. A substantial body of evidence supports the centralization of complex surgical procedures in specialized high‐volume centres, where greater procedural experience is associated with improved outcomes and lower complication rates [9, 26, 35]. This relationship is particularly relevant for PAO, a technically demanding procedure with a well‐established learning curve and demonstrated volume‐outcome relationship [2, 28, 32]. The increasing specialization and evolving indications of PAO further underscore the importance of surgical expertise and appropriate patient selection [31]. The current results suggest that the potential disadvantages associated with longer travel distances do not adversely affect short‐term outcomes after PAO. Consequently, these findings support the feasibility of concentrating PAO procedures in specialized centres in order to maintain surgical expertise and procedural volume while ensuring excellent clinical outcomes for patients, regardless of travel distance.

## LIMITATIONS

Several limitations should be considered when interpreting the findings of the present study.

First, the analysis is based on short‐term follow‐up data. Although meaningful improvements in PROMs can be detected within the first postoperative years, longer‐term follow‐up is required to determine whether the observed findings remain stable over time and whether travel distance influences survivorship, reoperation rates or long‐term functional outcomes.

Second, while follow‐up rates were high for most PROMs (approximately 80%–90%), lower follow‐up rates were observed for the HHS and PMA. This was primarily attributable to missing clinical examinations required for calculation of the HHS. Furthermore, the clinical parameters necessary for the HHS assessment were collected only at the 12‐ and 24‐month follow‐up visits. Similarly, PMA scores were obtained almost exclusively at these time points, resulting in fewer available observations compared with the other PROMs.

In addition, the G‐FJS was incorporated into the underlying hip registry after initiation of patient enrolment. Consequently, a substantial proportion of patients lacked either baseline G‐FJS measurements or corresponding follow‐up assessments. Owing to the resulting limited sample size, findings related to the G‐FJS should be interpreted with caution.

Third, several of the PROMs used in this study were originally developed and validated in older patient populations, particularly those undergoing arthroplasty [5, 7, 12, 13, 19]. In contrast, the present cohort had a mean age of approximately 30 years. Although these instruments are widely used in hip preservation research and demonstrated responsiveness in the current study, they may not fully capture all aspects of function and activity relevant to a younger and more active patient population.

Furthermore, the 80.5 km cutoff was derived from previous literature predominantly from studies conducted in the United States and may not represent a universally meaningful threshold for travel burden in other healthcare systems or geographic settings. However, sensitivity analysis treating travel distance as a continuous variable yielded consistent results suggesting that the findings were not dependent on the predefined cutoff.

Complications treated at external institutions may have been underreported, particularly among patients living farther from the treating centre, potentially resulting in differential under‐ascertainment in the long‐distance group.

Finally, the study was conducted at a single high‐volume hip preservation centre and included procedures performed by a single experienced surgeon. While this may limit generalizability to other institutions, the high procedural volume and standardized surgical and perioperative management reduce variability and strengthen the internal validity of the study.

## CONCLUSION

Travel distance to a specialized hip preservation centre was not significantly associated with differences in short‐term outcomes following PAO for DDH. Patients travelling more than 80.5 km (50 mi) showed no significant differences in patient‐reported outcomes, rates of achieving MCID and PASS or complication rates compared with patients residing closer to the treating centre.

These findings support the feasibility of centralized PAO care in specialized high‐volume centres, while the potential benefits of centralization itself are supported by the broader evidence on surgical expertise and procedural volume.

## AUTHOR CONTRIBUTIONS

**Quentin Karisch** and **Sufian S. Ahmad**: Formulation of the study design; statistical analysis; manuscript writing and revision. **Justus Stamp, Chiara Heller, Marco Haertlé** and **Henning Windhagen**: Graphics, manuscript writing and revision.

## CONFLICT OF INTEREST STATEMENT

The authors declare no conflicts of interest.

## ETHICS STATEMENT

This study was conducted in accordance with the principles of the Declaration of Helsinki. Ethical approval was obtained from the local ethics committee of Hannover Medical School (10450_BO_K_2022). The study was conducted at the Orthopaedic Department, Hannover Medical School, Anna‐von‐Borries‐Strasse 1‐7, 30625 Hannover, Germany. Written informed consent was obtained.

## Supporting information

Supporting file 1.

Supporting file 2.

## Data Availability

Raw data are available upon request.
