# Supplementary material for: Does travel distance influence short‐term outcome after periacetabular osteotomy?—A cohort study
Source: J Exp Orthop. 2026 Sep 24;13(3):e70912. doi: 10.1002/jeo2.70912 (PMC13604035; doi:10.1002/jeo2.70912)
Supplement: Supplementary file 1 [file JEO2-13-e70912-s001.docx]

**Supplementary Table S2:** Multivariable linear regression analyses for postoperative patient-reported outcome measures using travel distance as a continuous variable

| **Predictor** | **UCLA**  **β (95% CI, p)** | **HOOS-PS**  **β (95% CI, p)** | **WOMAC**  **β (95% CI, p)** | **iHOT-12**  **β (95% CI, p)** | **HHS**  **β (95% CI, p)** | **mHHS**  **β (95% CI, p)** | **G-FJS**  **β (95% CI, p)** | **PMA**  **β (95% CI, p)** |
| --- | --- | --- | --- | --- | --- | --- | --- | --- |
|  |  |  |  |  |  |  |  |  |
| ***Travel distance (per km)*** | 0.000  (−0.002 to 0.001, p=0.734) | 0.003  (−0.012 to 0.019, p=0.658) | −0.003  (−0.016 to 0.011, p=0.684) | 0.000  (−0.021 to 0.022, p=0.977) | 0.007  (−0.009 to 0.023, p=0.377) | <0.001  (−0.013 to 0.013, p=0.995) | −0.017  (−0.061 to 0.027, p=0.445) | <0.001  (−0.002 to 0.001, p=0.763) |
| ***Baseline PROM*** | 0.510  (0.401 to 0.618, **p<0.001**) | 0.377  (0.276 to 0.479, **p<0.001**) | 0.444  (0.354 to 0.534, **p<0.001**) | 0.485  (0.360 to 0.611, **p<0.001**) | 0.354  (0.208 to 0.499, **p<0.001**) | 0.357  (0.257 to 0.457, **p<0.001**) | 0.492  (0.229 to 0.756, **p<0.001**) | 0.129  (0.020 to 0.238, **p=0.021**) |
| ***LCEA*** | 0.050  (−0.009 to 0.109, p=0.093) | −0.509  (−1.134 to 0.117, p=0.110) | 0.461  (−0.121 to 1.042, p=0.120) | −0.772  (−1.655 to 0.110, p=0.086) | 0.197  (−0.494 to 0.888, p=0.574) | −0.350  (−0.860 to 0.161, p=0.178) | 0.049  (−1.497 to 1.595, p=0.950) | −0.026  (−0.094 to 0.043, p=0.459) |
| ***EI*** | 5.403  (−0.082 to 10.888, p=0.054) | −6.726  (−64.476 to 51.025, p=0.819) | −0.198  (−53.614 to 53.217, p=0.994) | −13.837  (−95.128 to 67.453, p=0.738) | 72.835  (7.599 to 138.071, p=0.029) | 24.790  (−22.469 to 72.050, p=0.303) | 121.655  (−26.530 to 269.840, p=0.106) | 1.790  (−4.769 to 8.350, p=0.591) |

**Abbreviations:** UCLA=University of California and Los Angeles activity scale; HOOS-PS=Hip Disability and Osteoarthritis Outcome Score–Physical Function Short Form; WOMAC=Western Ontario and McMaster Universities Osteoarthritis Index; G-FJS=German Forgotten Joint Score; iHOT-12=International Hip Outcome Tool–12; HHS=Harris Hip Score; mHHS=Modified Harris Hip Score; PMA=Postel–Merle d’Aubigné score; β=unstandardized regression coefficient; CI=confidence interval; p=p-value; PROM=patient-reported outcome measure; LCEA=lateral center-edge angle; EI=extrusion index.
