# Supplementary material for: Does travel distance influence short‐term outcome after periacetabular osteotomy?—A cohort study
Source: J Exp Orthop. 2026 Sep 24;13(3):e70912. doi: 10.1002/jeo2.70912 (PMC13604035; doi:10.1002/jeo2.70912)
Supplement: Supplementary file 2 [file JEO2-13-e70912-s002.docx]

**Supplementary Table S1:** Multivariable linear regression analyses for postoperative patient-reported outcome measures

| **Predictor** | **UCLA**  **β (95% CI, p)** | **HOOS-PS**  **β (95% CI, p)** | **WOMAC**  **β (95% CI, p)** | **iHOT-12**  **β (95% CI, p)** | **HHS**  **β (95% CI, p)** | **mHHS**  **β (95% CI, p)** | **G-FJS**  **β (95% CI, p)** | **PMA**  **β (95% CI, p)** |
| --- | --- | --- | --- | --- | --- | --- | --- | --- |
|  |  |  |  |  |  |  |  |  |
| ***Travel-distance group*** | −0.040  (−0.451 to 0.370, p=0.847) | 3.095  (−1.250 to 7.441, p=0.162) | −0.141  (−3.975 to 3.693, p=0.942) | 2.417  (−3.621 to 8.455, p=0.431) | −0.002  (−3.677 to 3.673, p=0.999) | 0.068  (−3.446 to 3.582, p=0.970) | −6.523  (−19.523 to 6.476, p=0.321) | −0.105  (−0.550 to 0.340, p=0.643) |
| ***Baseline PROM*** | 0.508  (0.400 to 0.616, **p<0.001**) | 0.379  (0.277 to 0.480, **p<0.001**) | 0.445  (0.354 to 0.535, **p<0.001**) | 0.485  (0.360 to 0.611, **p<0.001**) | 0.573  (0.456 to 0.691, **p<0.001**) | 0.357  (0.257 to 0.456, **p<0.001**) | 0.484  (0.220 to 0.748, **p<0.001**) | 0.131  (0.021 to 0.241, **p=0.020**) |
| ***LCEA*** | 0.051  (−0.008 to 0.110, p=0.093) | −0.479  (−1.102 to 0.145, p=0.132) | 0.468  (−0.114 to 1.050, p=0.114) | −0.743  (−1.626 to 0.140, p=0.099) | −0.162  (−0.698 to 0.374, p=0.552) | −0.349  (−0.861 to 0.163, p=0.180) | −0.021  (−1.578 to 1.537, p=0.979) | −0.027  (−0.095 to 0.042, p=0.446) |
| ***EI*** | 5.490  (0.035 to 10.946, **p=0.049**) | −7.293  (−64.522 to 49.935, p=0.802) | 0.763  (−52.469 to 53.995, p=0.977) | −13.328  (−94.233 to 67.578, p=0.746) | 27.226  (−22.201 to 76.653, p=0.279) | 24.805  (−22.296 to 71.907, p=0.301) | 128.460  (−16.168 to 273.089, p=0.081) | 1.815  (−4.721 to 8.352, p=0.585) |

**Abbreviations:** UCLA=University of California and Los Angeles activity scale; HOOS-PS=Hip Disability and Osteoarthritis Outcome Score–Physical Function Short Form; WOMAC=Western Ontario and McMaster Universities Osteoarthritis Index; G-FJS=German Forgotten Joint Score; iHOT-12=International Hip Outcome Tool–12; HHS=Harris Hip Score; mHHS=Modified Harris Hip Score; PMA=Postel–Merle d’Aubigné score; β=unstandardized regression coefficient; CI=confidence interval; p=p-value; PROM=patient-reported outcome measure; LCEA=lateral center-edge angle; EI=extrusion index.
